# Case Report: Integrated traditional Chinese and Western medicine in the outpatient management of diabetic foot gangrene complicating uremia

**DOI:** 10.3389/fendo.2025.1638086

**Published:** 2025-08-27

**Authors:** Can Hu, Jian Chen

**Affiliations:** ^1^ Department of Vascular Diseases, Shanghai TCM-Integrated Hospital, Shanghai, China; ^2^ Institute of Vascular Anomalies, Shanghai TCM-Integrated Hospital, Shanghai University of Traditional Chinese Medicine, Shanghai, China; ^3^ Anhui Province Rural Revitalization Collaborative Technical Service Center, Huangshan University, Huangshan, China; ^4^ Department of Public Health, International College, Krirk University, Bangkok, Thailand

**Keywords:** diabetic foot, gangrene, uremia, traditional Chinese medicine, inflammation

## Abstract

Diabetic foot gangrene (DFG) in uremic patients presents profound management challenges due to immune dysfunction and impaired tissue repair. We report on a 65-year-old male patient with end-stage renal disease undergoing peritoneal dialysis, presenting with progressive right foot gangrene that was unresponsive to conventional treatment. Clinical evaluation indicated extensive gangrene affecting the first and second toes, elevated inflammatory markers (C-reactive protein at 95.57 mg/L and fibrinogen at 8.18 g/L), and cardiovascular compromise (brain natriuretic peptide at 4869 pg/mL). Lower limb computed tomography angiography confirmed severe atherosclerosis. An integrated protocol combining Yanghe Sijunzi Decoction with meticulous debridement and localized analgesia was implemented over 7 months (18 outpatient visits). This approach resulted in complete ulcer healing by October 2024, with the resolution of gangrene, an 87% reduction in CRP levels (to 12 mg/L), normalization of coagulation markers, and preservation of foot function—thus preventing amputation. Patient-reported outcomes, including pain scores and sleep duration gradually improved as healing advanced from 23 March 2024. The total treatment costs amounted to 25,000 CNY, with the patient’s share being 5,000 CNY, demonstrating cost-efficiency. This case highlights Traditional Chinese Medicine’s potential as an immunomodulatory adjuvant in uremia-associated DFG, modulating inflammation and promoting tissue regeneration in high-risk patients.

## Introduction

Uremia, a condition characterized by the accumulation of waste products in the blood due to renal failure, poses significant health challenges, particularly when compounded by diabetes mellitus (1). Diabetes is a leading cause of end-stage renal disease (ESRD), and patients with both uremia and diabetes are at an increased risk for complications such as diabetic foot ulcers and subsequent gangrene. The pathophysiology underlying diabetic foot ulcers involves a combination of neuropathy, ischemia, and infection, which can lead to severe tissue damage and necessitate amputation if not managed effectively. The clinical presentation of diabetic foot ulcers can vary, but common symptoms include pain, discoloration, and gangrene of the affected areas, often accompanied by systemic signs of infection and metabolic derangements (2).

The management of diabetic foot gangrene in the context of uremia is particularly complex due to the interplay of multiple comorbidities, including cardiovascular disease and impaired renal function. Current treatment strategies often involve a combination of surgical intervention, such as debridement, and pharmacological therapies aimed at controlling blood glucose levels and preventing infection. However, the efficacy of these approaches can be limited by the patient’s overall health status and the presence of other complications, such as peripheral vascular disease (3).

This case report presents a 65-year-old male patient with uremia and diabetic foot gangrene, highlighting the challenges and intricacies of managing such a rare and severe condition. The importance of this case lies in its potential to serve as a differential diagnosis for patients presenting with similar symptoms, particularly in those with a background of diabetes and renal impairment. The integration of traditional Chinese medicine with conventional Western treatments offers a unique perspective on managing this complex condition, emphasizing the need for a holistic approach to patient care.

## Case presentation

### Introduction to the clinical case

A 65-year-old male patient with a BMI of 21 and an HbA1c level of 7.1 presented to our outpatient department on March 12, 2024. His primary complaint was gangrene, pain, and ulceration affecting multiple digits on his right foot, which had persisted for over three months. Additionally, he reported experiencing generalized fatigue, limb coldness, and a poor appetite. The patient had a history of hypertension for over 10 years, diabetes mellitus for 8 years, and uremia for more than 3 years, for which he had been undergoing regular peritoneal dialysis (PD) four times a day. Additionally, he was on multiple medications, including spironolactone, clopidogrel, isosorbide mononitrate, rosuvastatin, sacubitril valsartan, linagliptin, enteric-coated aspirin, sevelamer carbonate, epoetin alfa, vitamin D soft capsules, nifedipine, and Jinshuibao tablets. His condition was regularly evaluated at a tertiary hospital’s nephrology department.

On January 15, 2024, the patient was admitted to the nephrology department at a local hospital for peritoneal dialysis, blood pressure and blood sugar control, heart function improvement, and various other treatments. However, right lower limb angiography (DSA) was performed, which revealed severe arterial occlusion on January 18. During this hospitalization, he was also diagnosed with cognitive dysfunction and had elevated troponin I levels, leading to a consultation with the cardiology department. He was subsequently diagnosed with multiple brain infarctions (CT on January 20). After a series of treatments, including angioplasty (PTA) of the right lower limb arteries, the patient was discharged on January 28, but his resting pain (based on the Numeric Rating Scale), foot gangrene, and ulceration continued to progress. A re-admission to a different hospital on February 14, 2024, led to further testing and conservative management of his lower limb symptoms, but no significant improvement was noted.

### Clinical assessment and diagnostic work-up

Upon his visit to our outpatient clinic, the patient appeared lethargic and physically weak, with a yellowish, dull complexion and minimal response to questions. His right foot was swollen with a dark red discoloration and cold temperature. The first digit (big toe) showed extensive gangrene covered with black gangrene, and the second toe showed partial gangrene with exposed tissue and granulation. There was also joint destruction in the affected areas. On palpation, the distal extremities were cold, and the patient had slight cognitive impairment.

Several laboratory and imaging tests were conducted. Blood tests revealed mild anemia (hemoglobin 115 g/L), elevated CRP (95.57 mg/L), and signs of chronic kidney dysfunction with a serum creatinine of 748 µmol/L. Blood gas analysis showed an increased carbon dioxide pressure (57.8 mmHg) and decreased potassium levels (3.2 mmol/L). Inflammatory markers were notable with elevated BNP (4869 pg/ml) and troponin I (0.18 ng/ml), suggesting significant cardiovascular involvement. Computed tomography angiography (CTA) of the lower limbs demonstrated extensive atherosclerotic changes, with stenosis and occlusion in the iliac, femoral, and tibial arteries. Further tests, including DSA, confirmed a lack of significant arterial occlusion in the right lower limb, which helped rule out acute arterial thrombosis.

Based on the patient’s comprehensive clinical presentation, his medical history, and diagnostic findings, the differential diagnosis included diabetic foot syndrome, arteriosclerotic occlusion, uremic vasculopathy, and secondary infections. The patient was diagnosed with “gangrene” in traditional Chinese medicine (TCM), characterized by “spleen and kidney yang deficiency” and “phlegm and dampness obstructing the collaterals.” In Western medicine, the diagnoses included diabetic foot, arteriosclerotic occlusive disease, uremia, coronary heart disease post-stent placement, and chronic heart failure.

### Therapeutic approach and intervention

Given the complex nature of the patient’s condition, a multidisciplinary treatment approach was employed. On March 12, 2024, the patient was prescribed Yang He Tang combined with Si Jun Zi Tang, which contained raw astragalus membranaceus, raw atractylodes macrocephala, coix seed, white poria, honey-fried ephedra sinica, cinnamon twig, deer antler gelatin, white mustard seed, prepared rehmannia glutinosa, dried tangerine peel, corydalis rhizome, raw white peony root, propolis, processed rhubarb, and forsythia suspensa. The patient takes one packet per day for seven consecutive days, totaling seven packets. Local wound management included debridement of necrotic tissue, with the first session focusing on removing black gangrene from the first toe and draining any accumulated fluid. The wound was dressed with materials that promoted fluid drainage while minimizing irritation, using biocompatible disinfectants with antimicrobial properties.

Pain management focused on avoiding oral analgesics due to the patient’s underlying conditions. Instead, local application of lidocaine gel was used to alleviate discomfort around the wound edges. At the first follow-up on March 18, the patient’s general condition showed noticeable improvement. The appetite increased, and his fatigue and cold extremities improved. The first and second toes did not exhibit further expansion of gangrene, and wound granulation began to form. The treatment regimen remained the same, with slight modifications based on the patient’s response.

By March 26, the patient’s condition had further improved, although he had sustained a new injury to his right foot, which temporarily worsened the ulceration. Local debridement continued, and oral antibiotics (clindamycin) were initiated for infection control. The patient also experienced a reduction in systemic symptoms, including cold extremities and foot pain. The prescribed traditional Chinese medicine continued, with a focus on stabilizing his overall condition and supporting wound healing.

### Long-term follow-up and outcome

**Figure 1:**
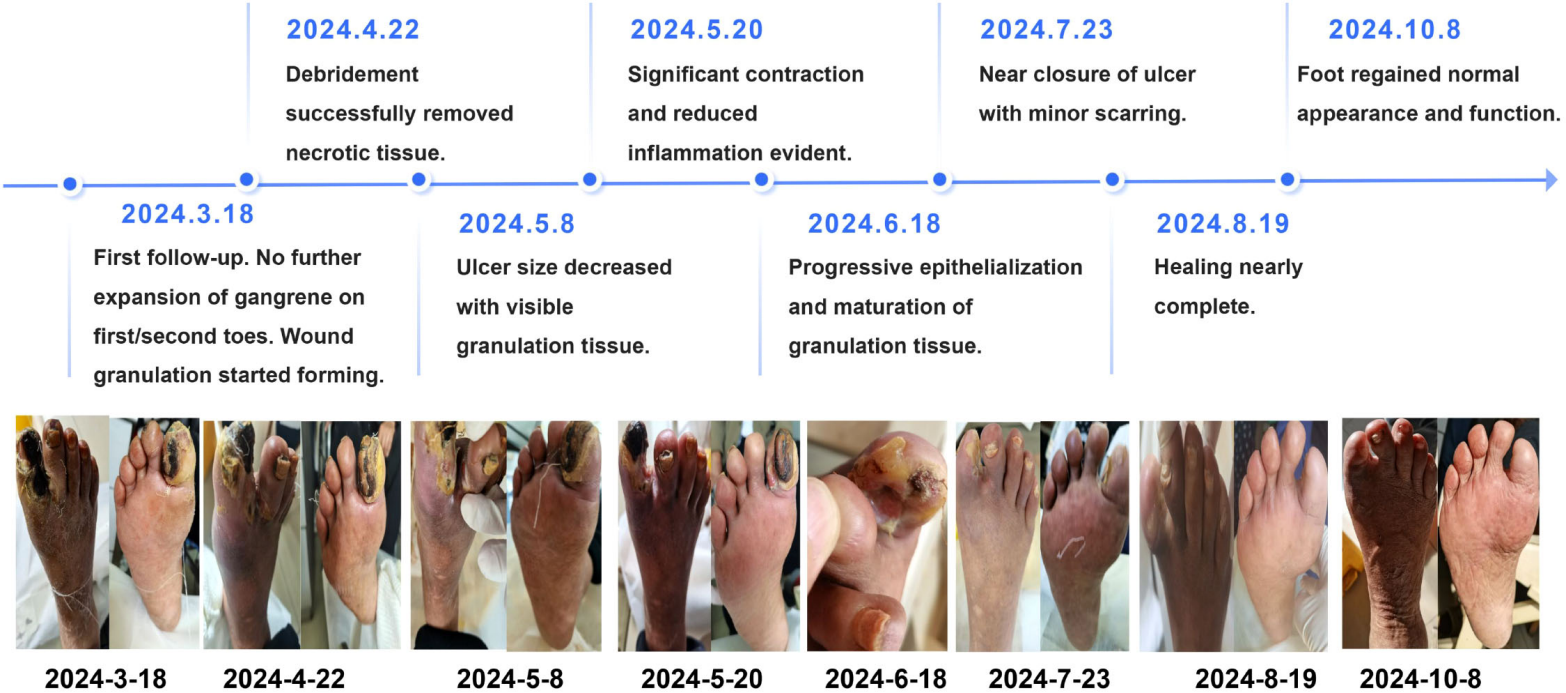
The treatment process of a patient with diabetic foot gangrene from March 18, 2024 to October 8, 2024.

The patient continued with regular follow-up visits every two weeks, receiving treatment with traditional Chinese medicine and wound care. From March 23, a consistent downward trend in pain scores and increased trend in sleeping time were observed over time, indicating a significant reduction in pain severity with clinical management. By April 8, significant progress was observed, with less swelling, reduced pain, and improved circulation in the lower extremities. Granulation tissue in the ulcerated areas was pink and healthy, signaling progress in wound healing. **Figure 1** depicting the ulcer healing process from March 18, 2024, to October 8, 2024, revealed critical milestones. Initially, the foot exhibited severe infection and gangrene. By April 22, debridement had successfully removed necrotic tissue. By May 8, the ulcer size decreased with visible granulation tissue, and by May 20, significant contraction and reduced inflammation were evident. Progressive epithelialization and maturation of granulation tissue were noted by June 18, followed by near closure of the ulcer with minor scarring by July 23. By August 19, healing was nearly complete, and by October 8, the foot regained normal appearance and function, underscoring the efficacy of debridement and advanced wound care.

**Figure 2:**
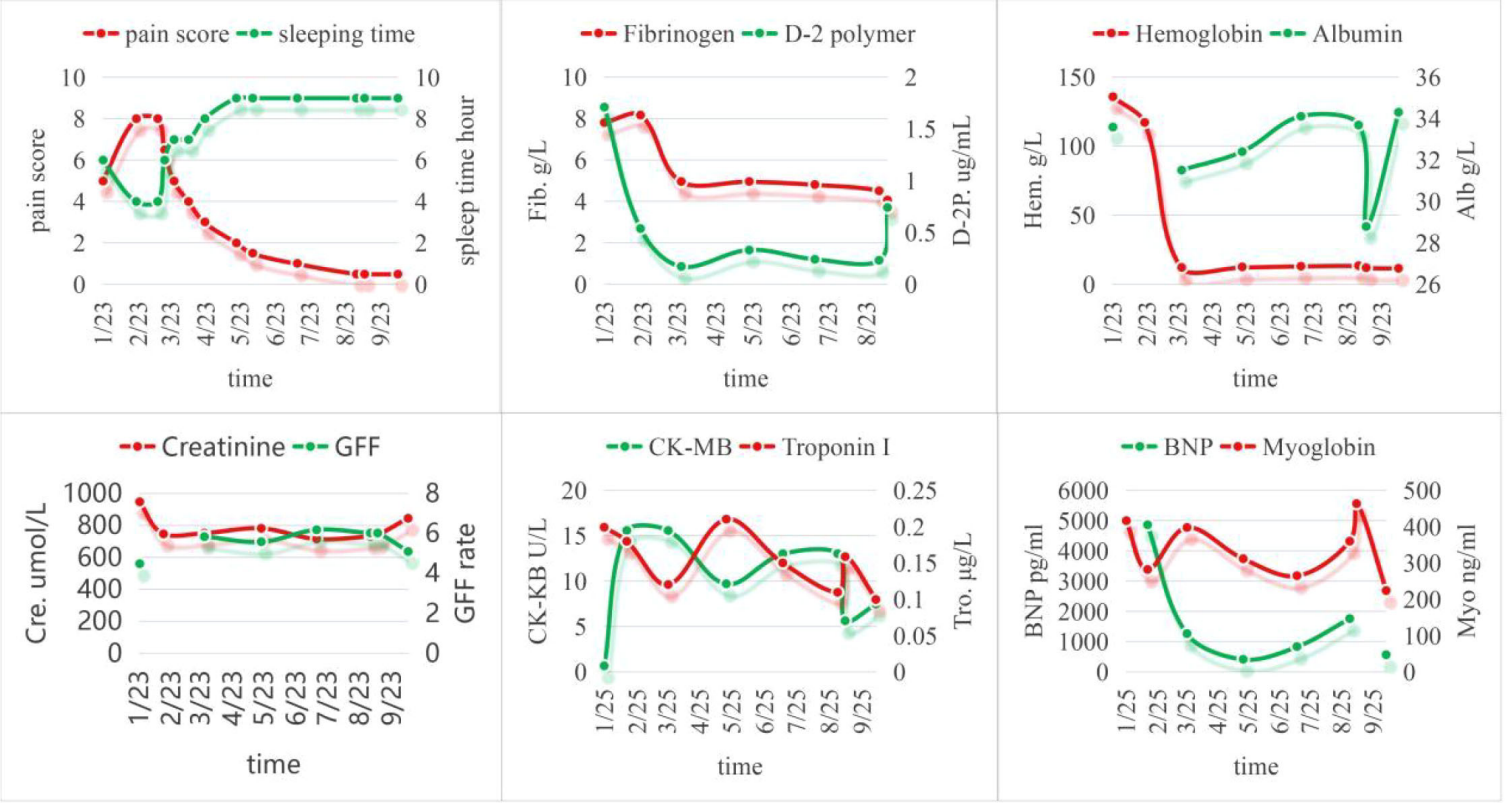
Temporal trends of clinical biomarkers and indicators in a patient with diabetic foot gangrene. Quality of life: pain score and sleeping time recorded at each follow-up visit. Coagulation and fibrinolysis systems: fibrinogen and D-2 polymer. Nutrition: hemoglobin and albumin. Renal function: creatinine and glomerular filtration rate (GFR). Heart function: creatine kinase-MB (CK-MB), troponin I, Brain Natriuretic Peptide (BNP) and myoglobin.

By May 6, the ulceration on the first toe showed signs of healing, and the pain had diminished substantially. The patient’s nutritional status, including albumin levels, improved, reflecting a more stable condition. Concurrent biomarker trends mirrored clinical improvements: fibrinogen and D-dimer levels declined steadily, indicating reduced inflammation and coagulation activity. Hemoglobin and albumin levels, initially low, recovered progressively, reflecting improved nutrition. Renal function markers (creatinine and GFR) remained stable, while transient elevations in cardiac markers (CK-MB, troponin I) suggested minor stress events, though they normalized over time. Patient-reported outcomes, including pain scores and sleep duration, fluctuated initially but gradually improved as healing advanced (**Figure 2**).

In subsequent visits, the patient continued to make steady progress. By June 3, the first toe was almost healed, and there was noticeable reduction in the gangrene of the second toe. Local debridement was performed, including the removal of any remaining necrotic tissue. The patient’s condition remained stable through July and August, with significant improvements in foot temperature, pain, and swelling. However, the second toe showed mild deformity, which was managed conservatively, with advice to reduce weight-bearing on the affected foot.

By September 23, the patient reported no pain or cold sensations in the affected foot, and the first toe ulcer had nearly healed, with new tissue forming well. Although the patient continued to experience mild visual disturbances, the overall prognosis was promising. The continued use of traditional Chinese medicine and tailored wound care strategies helped support healing and improve the patient’s quality of life.

### Cost analysis of outpatient-dominated management

During the seven-month treatment period from March 12 to October 8, the patient incurred total medical expenses of approximately 25,000 CNY (Chinese Yuan) across 18 outpatient visits. This included: less than 5,000 CNY for five blood tests; 140 CNY for two foot radiographs; 660 CNY for two lower limb arterial Doppler ultrasound examinations; approximately 2,000 CNY for Western medications; and about 14,000 CNY for Traditional Chinese Medicine (TCM) over six months. Additional costs comprised approximately 1,700 CNY for six physician-performed debridement procedures under local anesthesia and 700 CNY for follow-up wound dressing changes. The patient’s out-of-pocket expenditure was approximately 5,000 CNY. Notably, the only hospitalization during this period occurred on September 9 at the designated end-stage renal disease (ESRD) management hospital for assessment, which required no additional interventions.

## Discussion

The management of diabetic foot gangrene in patients with uremia represents a formidable clinical challenge due to the convergence of multiple comorbidities, including neuropathy, peripheral vascular disease (PVD), impaired immunity, and metabolic dysregulation. However, there was limited published evidence for successful outpatient-based integrated Traditional Chinese and Western Medicine approaches in managing severe DFG in ESRD patients, particularly those who have been refractory to conventional care. Moreover, patients with ESRD are at increased risk of DFU and have a higher mortality rate (4). Therefore, the importance of ongoing comprehensive management will be emphasized. This includes strict glycemic control, meticulous foot care, consistent adherence to peritoneal dialysis, and continuous monitoring for early signs of recurrence or other diabetes- and uremia-related complications. This case highlights the intricate interplay between diabetes, CKD, and lower extremity complications, emphasizing the need for a holistic and multidisciplinary approach.

DFUs affect 6.3% of the global diabetic population, with higher prevalence in males, type 2 diabetes. gangrene, a severe progression of DFUs, occurs in 0.19% of cases and is strongly associated with prolonged diabetes duration, hypertension, and PVD (5). Notably, CKD exacerbates DFU risks, with 39.3% of DFU patients concurrently suffering from CKD (6). In ESRD, dialysis further amplifies the risk of DFUs by fourfold due to uremic toxin accumulation, proteinuria-induced edema, and immunosuppression (7). Our patient’s clinical profile—long-standing diabetes, hypertension, ESRD on peritoneal dialysis, and multi-digit gangrene—exemplifies this high-risk cohort, where delayed wound healing and systemic infections are common.

Current guidelines prioritize aggressive infection control, surgical debridement, vascular assessment, and glycemic management. However, in uremic patients, therapeutic efficacy is often compromised. For instance, antibiotic pharmacokinetics are altered in renal failure, necessitating dose adjustments to avoid toxicity. Additionally, peripheral arterial disease (PAD), present in 28.7% of DFU patients, limits the success of revascularization and increases amputation risks (8). In this case, the patient’s ischemia and recurrent infections likely contributed to poor response to initial debridement and antimicrobial therapy, underscoring the limitations of standard protocols in comorbid populations.

Effective management of such complex cases necessitates collaboration among nephrologists, vascular surgeons, endocrinologists, and wound care specialists. Multidisciplinary care (MDC) models reduce mortality, hospitalization rates, and temporary dialysis catheter use in CKD patients by optimizing medication adherence, dietary management, and vascular access planning (9). In this case, regular evaluations by nephrology and surgical teams, coupled with dialysis compliance, were critical in mitigating uremic complications and stabilizing metabolic parameters. MDC also facilitates timely transitions between conservative and surgical interventions, as seen in the patient’s staged debridement and subsequent vascular reassessment.

This case underscores the complexity of managing diabetic foot gangrene in uremia and advocates for integrative, patient-centered strategies. While conventional therapies address acute complications, TCM adjuncts and MDC models optimize long-term outcomes by targeting metabolic, inflammatory, and vascular pathways. The TCM is composed of Yanghe Decoction (YH) and Sijunzi Decoction (SJZD). YH has demonstrated anti-inflammatory effects, notably by improving NLRP3 inflammasome and immune dysregulation (10). It has also been shown to modulate the JAK/STAT pathway, reduce Myeloid-Derived Suppressor Cells (MDSCs), interleukin-6 (IL-6), and transforming growth factor-beta (TGF-β), while increasing interferon-gamma (IFN-γ), Natural Killer T Cells (NKTs), and CD4+ T cells (11). SJZD, on the other hand, is known for its ability to alleviate inflammation, modulate immune responses, regulate intestinal flora, and facilitate mucosal repair (12). These actions are pertinent to mitigating the chronic inflammation and immune dysfunction prevalent in DFG patients, especially those with uremia.

Amputations are a frequent and severe outcome, with at least half of all amputations occurring in individuals with diabetes. Between 14% and 24% of DFU patients require major or minor lower limb amputation (13). Furthermore, ESRD patients face a significantly increased risk of DFU and subsequent amputation, and up to nine-fold compared to non-diabetic individuals (4). This successful limb salvage in a highly complex patient who was refractory to initial conventional care underscores the potential advantages of the integrated approach. While direct randomized controlled trials comparing integrative DFG treatment in uremic patients may be limited, existing evidence supports the efficacy of TCM therapy in improving wound healing in DFU (14). Moreover, the integration of TCM and Western Medicine has demonstrated enhanced overall effectiveness rates in diabetic kidney disease (15). This case report, therefore, serves as crucial preliminary evidence, suggesting that this integrative outpatient strategy warrants further investigation as a viable alternative in this vulnerable patient population.

Pain management emerges as a critical component of the treatment strategy. Effective analgesia not only improves patient comfort but also enhances participation in rehabilitation efforts. The use of localized anesthetic agents, as exemplified in this case, can provide immediate relief while minimizing systemic side effects. Literature supports the need for personalized pain management strategies that are both rapid and sustainable, which can significantly influence the patient’s overall treatment trajectory (16). Moreover, the timing and methodology of surgical debridement are critical considerations in managing diabetic foot gangrene. The findings of this case indicate that timely intervention, guided by clinical assessments and imaging results, is essential for optimizing patient outcomes.

Research indicates that the average length of hospital stay for DFU in China is approximately 21 days, with an average cost of around $8672 (approximately ¥62,000 at current exchange rates) (17). Furthermore, the average cost of amputation in China is reported to be ¥45,951.78 (18). Hospitalization costs for uremia average around $2150 (approximately ¥15,000) (19), and the monthly cost of hemoperfusion for ESRD can be around $333 (approximately ¥2,400) (20). The patient’s total outpatient cost of 25,000 CNY for complete healing and limb salvage is significantly more cost-efficient when compared to the average costs associated with DFU hospitalization or, more critically, amputation. This comparison underscores the substantial economic value of the outpatient-dominated integrative approach, demonstrating its potential to reduce the significant financial burden associated with severe diabetic complications and ESRD.

Limitations of this case report should be acknowledged. First, as a single-patient case report, the findings may not be generalizable to broader populations. Second, although improvements in inflammation and immune-related symptoms were observed, cytokine profiling or immune panel testing was not performed, which limits the ability to confirm the immunomodulatory effects of the TCM interventions. Future studies with larger cohorts and detailed immunological assessments are warranted to validate these findings.

In conclusion, the patient’s successful recovery from severe diabetic foot ulcers and gangrene demonstrates the efficacy of an integrative treatment approach combining traditional Chinese medicine with modern wound management. Regular follow-ups, wound care, and tailored pharmacological therapies were crucial in improving the patient’s condition and preventing further complications, offering valuable insights into managing complex, multi-system diseases in older patients with comorbidities.

## Data Availability

The original contributions presented in the study are included in the article/supplementary material. Further inquiries can be directed to the corresponding author.

## References

[1] Bakinowska E Olejnik-Wojciechowska J Kielbowski K Skoryk A Pawlik A. Pathogenesis of sarcopenia in chronic kidney disease-the role of inflammation, metabolic dysregulation, gut dysbiosis, and microRNA. Int J Mol Sci. (2024) 25:8474. doi: 10.3390/ijms25158474, PMID: 39126043 PMC11313360

[2] Lan NSR Dwivedi G Fegan PG Game F Hamilton EJ. Unravelling the cardio-renal-metabolic-foot connection in people with diabetes-related foot ulceration: a narrative review. Cardiovasc Diabetol. (2024) 23:437. doi: 10.1186/s12933-024-02527-1, PMID: 39696281 PMC11657306

[3] Kaminska M Kalucka U Babickova J Benedyk-Machaczka M Skandalou E Grant MM. Bradykinin's carbamylation as a mechanistic link to impaired wound healing in patients with kidney dysfunction. BMC Biol. (2025) 23:76. doi: 10.1186/s12915-025-02187-x, PMID: 40075424 PMC11905624

[4] Bonnet JB Sultan A. Narrative review of the relationship between CKD and diabetic foot ulcer. Kidney Int Rep. (2022) 7:381–8. doi: 10.1016/j.ekir.2021.12.018, PMID: 35257052 PMC8897302

[5] Al-Rubeaan K Al Derwish M Ouizi S Youssef AM Subhani SN Ibrahim HM. Diabetic foot complications and their risk factors from a large retrospective cohort study. PloS One. (2015) 10:e0124446. doi: 10.1371/journal.pone.0124446, PMID: 25946144 PMC4422657

[6] Liang J An H Hu X Gao Y Zhou J Gong X. Correlation between chronic kidney disease and all-cause mortality in diabetic foot ulcers: evidence from the 1999–2004 national health and nutrition examination survey (NHANES). Front Endocrinol (Lausanne). (2025) 16:1533087. doi: 10.3389/fendo.2025.1533087, PMID: 40162314 PMC11949790

[7] He Y Qian H Xu L Zhang S Gu X Gu J. Association between estimated glomerular filtration rate and outcomes in patients with diabetic foot ulcers: a 3-year follow-up study. Eur J Endocrinol. (2017) 177:41–50. doi: 10.1530/EJE-17-0070, PMID: 28424173

[8] Roberts RHR Davies-Jones GR Brock J Satheesh V Robertson GA. Surgical management of the diabetic foot: The current evidence. World J Orthop. (2024) 15:404–17. doi: 10.5312/wjo.v15.i5.404, PMID: 38835689 PMC11145970

[9] Arooj H Aman M Hashmi MU Nasir Z Zahid M Abbas J. The impact of nurse-led care in chronic kidney disease management: a systematic review and meta-analysis. BMC Nurs. (2025) 24:188. doi: 10.1186/s12912-025-02829-z, PMID: 39966917 PMC11837475

[10] Ma B Chen D Liu Y Zhao Z Wang J Zhou G. Yanghe decoction suppresses the experimental autoimmune thyroiditis in rats by improving NLRP3 inflammasome and immune dysregulation. Front Pharmacol. (2021) 12:645354. doi: 10.3389/fphar.2021.645354, PMID: 34234669 PMC8255388

[11] Mao D Feng L Gong H. The antitumor and immunomodulatory effect of yanghe decoction in breast cancer is related to the modulation of the JAK/STAT signaling pathway. Evid Based Complement Alternat Med. (2018) 2018:8460526. doi: 10.1155/2018/8460526, PMID: 30581487 PMC6276440

[12] Yang L Fang Z Zhu J Li X Yang B Liu H. The potential of Sijunzi decoction in the fight against gastrointestinal disorders: a review. Front Pharmacol. (2025) 16:1464498. doi: 10.3389/fphar.2025.1464498, PMID: 40103588 PMC11913818

[13] Wadhawan N Shibuya N Jupiter DC. The impact of minor amputation on occurrence of major amputation in patients with diabetic foot ulcers. J Foot Ankle Surg. (2025) S1067-2516:00183–8. doi: 10.1053/j.jfas.2025.05.013, PMID: 40618832

[14] Wang Y Cao HJ Wang LQ Lu CL Yan YQ Lu H. The effects of Chinese herbal medicines for treating diabetic foot ulcers: A systematic review of 49 randomized controlled trials. Complement Ther Med. (2019) 44:32–43. doi: 10.1016/j.ctim.2019.03.007, PMID: 31126573

[15] Cai R Li C Zhao Y Yuan H Zhang X Liang A. Traditional Chinese medicine in diabetic kidney disease: multifaceted therapeutic mechanisms and research progress. Chin Med. (2025) 20:95. doi: 10.1186/s13020-025-01150-w, PMID: 40598250 PMC12211247

[16] Yang Y Zhao B Wang Y Lan H Liu X Hu Y. Diabetic neuropathy: cutting-edge research and future directions. Signal Transduct Target Ther. (2025) 10:132. doi: 10.1038/s41392-025-02175-1, PMID: 40274830 PMC12022100

[17] Ge Q Zhou Y Liu Z. Analysis of pre-hospital delay in Chinese patients with diabetic foot ulcers: Based on 46 cases. Int Wound J. (2023) 20:2657–63. doi: 10.1111/iwj.14139, PMID: 36916307 PMC10410325

[18] Lu Q Wang J Wei X Wang G Xu Y Lu Z. Cost of diabetic foot ulcer management in China: A 7-year single-center retrospective review. Diabetes Metab Syndr Obes. (2020) 13:4249–60. doi: 10.2147/DMSO.S275814, PMID: 33204131 PMC7667006

[19] Feng J Xu K Shi X Xu L Liu L Wang F. Incidence and cost of haemolytic uraemic syndrome in urban China: a national population-based analysis. BMC Nephrol. (2022) 23:122. doi: 10.1186/s12882-022-02746-2, PMID: 35354386 PMC8969241

[20] Wang H Jin H Cheng W Qin X Luo Y Liu X. Cost-effectiveness analysis of hemodialysis plus hemoperfusion versus hemodialysis alone in adult patients with end-stage renal disease in China. Ann Transl Med. (2021) 9:1133. doi: 10.21037/atm-21-1100, PMID: 34430574 PMC8350641

